# Enucleation of a giant symptomatic gastric lipoma, a safe surgical approach

**DOI:** 10.1093/jscr/rjab087

**Published:** 2021-03-27

**Authors:** Rafaela Parreira, Tiago Rama, Teresa Eloi, Vítor Carneiro, Maria Inês Leite

**Affiliations:** General Surgery Department, Hospital do Divino Espírito Santo, EPE, Ponta Delgada, Portugal; General Surgery Department, Hospital do Divino Espírito Santo, EPE, Ponta Delgada, Portugal; General Surgery Department, Hospital do Divino Espírito Santo, EPE, Ponta Delgada, Portugal; Pathology Department, Hospital Divino Espírito Santo, EPE, Ponta Delgada, Açores, Portugal; General Surgery Department, Hospital do Divino Espírito Santo, EPE, Ponta Delgada, Portugal

## Abstract

Gastric lipomas are rare, representing 2–3% of all benign tumours of the stomach. Most of these stomach neoplasms are small and detected incidentally during endoscopic or radiology evaluations. Computed tomography is highly specific imaging for lipoma diagnosis. Endoscopy and endoscopic ultrasound are other important diagnostic modalities to confirm the diagnosis. Identifying typical features can avoid biopsy or surgery in asymptomatic patients. In patients with larger lesions, usually more than 2 cm, clinical presentation may encompass haemorrhage, abdominal pain, pyloric obstruction and dyspepsia. As a result of its extreme low incidence, treatment is not standardized, though it is widely accepted that a symptomatic tumour mandates resection. Here, we present the case of a 60-year-old female presenting with abdominal pain and recurrent vomiting due to a giant gastric lipoma (80 × 35 × 35 mm). The patient underwent laparotomy and an enucleation was performed.

## INTRODUCTION

Gastrointestinal (GI) lipomas are uncommon benign and slow-growing tumours composed of mature adipose tissue surrounded by a fibrous capsule. Although GI lipomas can occur in any part of the gastrointestinal tract, its most affected location is the colon, followed by the ileum and jejunum [1]. Gastric lipomas are rare, accounting for 2–3% of all benign gastric tumours and less than 1% of all gastric neoplasms [2, 3].

**Figure 1:**
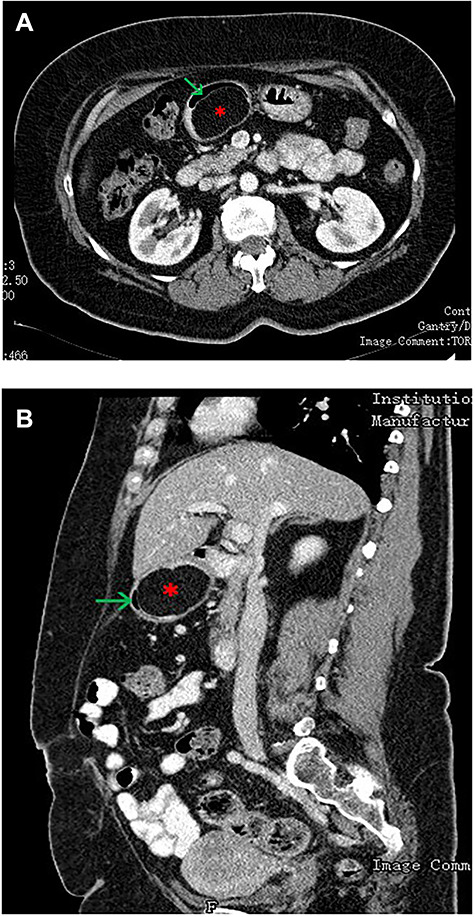
(**A**—coronal plane; **B**—sagittal plane): CT scan of the abdomen showing a large well-encapsulated and fat-attenuated submucosal lesion (8 × 3 cm) in the posterior wall of the gastric antrum, which causes lumen obstruction (^*^gastric lipoma, arrow—gastric lumen).

**Figure 2:**
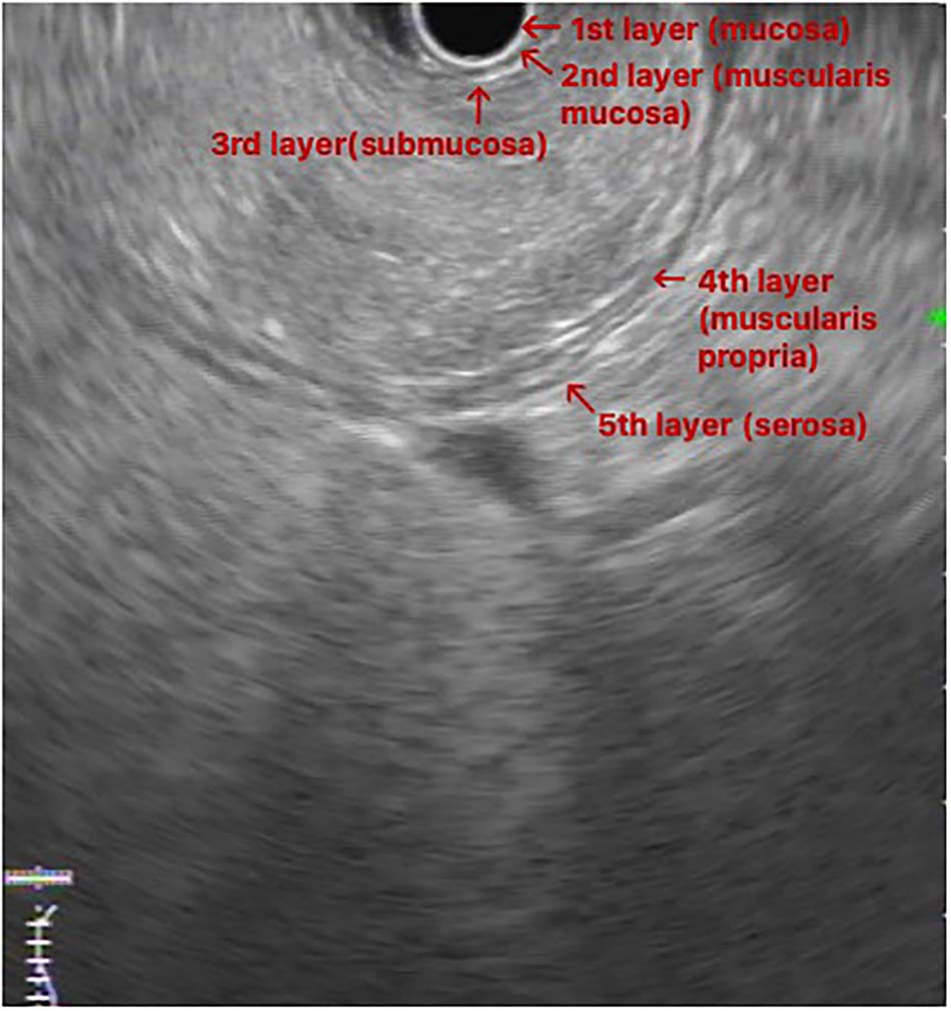
EUS image: homogeneous and hyperechoic lesion in submucosa layer.

Giant gastric lipomas (≥4 cm) are even more unusual [3]. The vast majority of gastric lipomas are small and discovered incidentally during endoscopic or radiology evaluations [4, 5]. The small asymptomatic lesions can be followed without intervention [6]. However, when symptoms occur, they are related to lipoma size, usually larger than 2 cm, and localisation near the pylorus. Clinical presentation may include upper gastrointestinal bleeding and gastrointestinal obstruction (epigastric pain, vomiting and dyspepsia) [6, 7]. Importantly, the treatment is not standardized, and available data are usually presented as case reports or small series [3].

## CASE REPORT

A 60-year-old female patient, with medical history of diabetes mellitus type 2, coronary artery disease, grade II obesity and dyslipidaemia, presented with complains of intermittent abdominal pain and postprandial vomiting over the past 6 months. The patient referred worsening symptoms in the last 3 months and an isolated episode of hematemesis, without hemodynamic repercussion or symptomatic anaemia. She denied weight loss, melena or change in bowel habits throughout this period. Clinical examination was unremarkable, and routine blood tests results showed values within normal range.

An upper GI endoscopy revealed a large subepithelial mass arising from the posterior wall of the gastric antrum, occupying more than 50% of the lumen, soft when pressed with biopsy forceps. The biopsies were non-diagnostic. Additionally, a contrast-enhanced computed tomography (CT) demonstrated a well-defined lesion of the antrum, measuring 8 × 3 cm, non-contrast enhancing and with uniform adipose density, highly suggestive of lipoma (Fig. 1). Endoscopic ultrasound (EUS) also showed a homogeneous and hyperechoic lesion limited to the submucosa (Fig. 2), supporting lipoma diagnosis.

**Figure 3:**
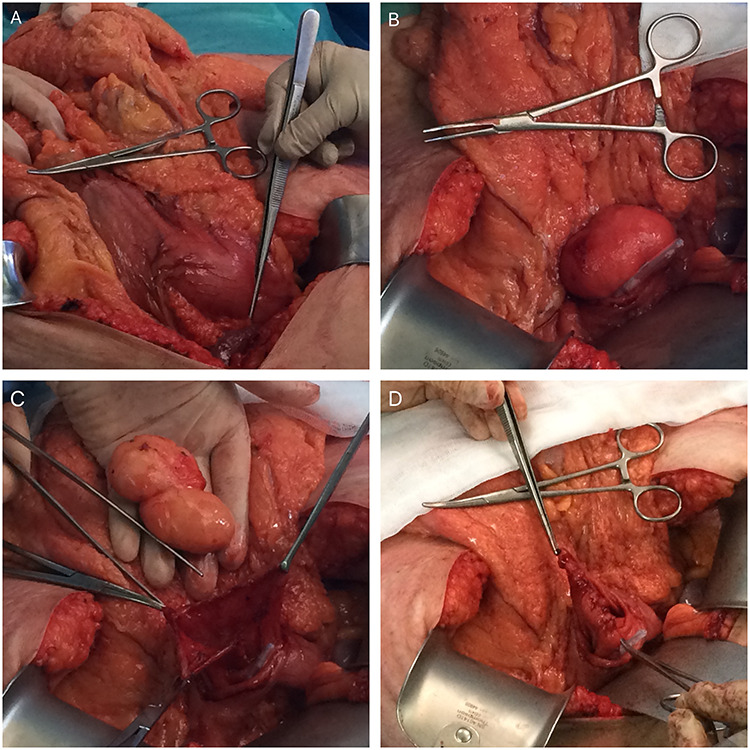
Intraoperative photos (**A**—mass-effect of the gastric lipoma; **B**—lipoma in the lumen of the stomach after anterior gastrotomy (slight mucosa ulceration); **C**—enucleated gastric lipoma (open mucosa); **D**—Sutured posterior wall mucosa after enucleation).

**Figure 4:**
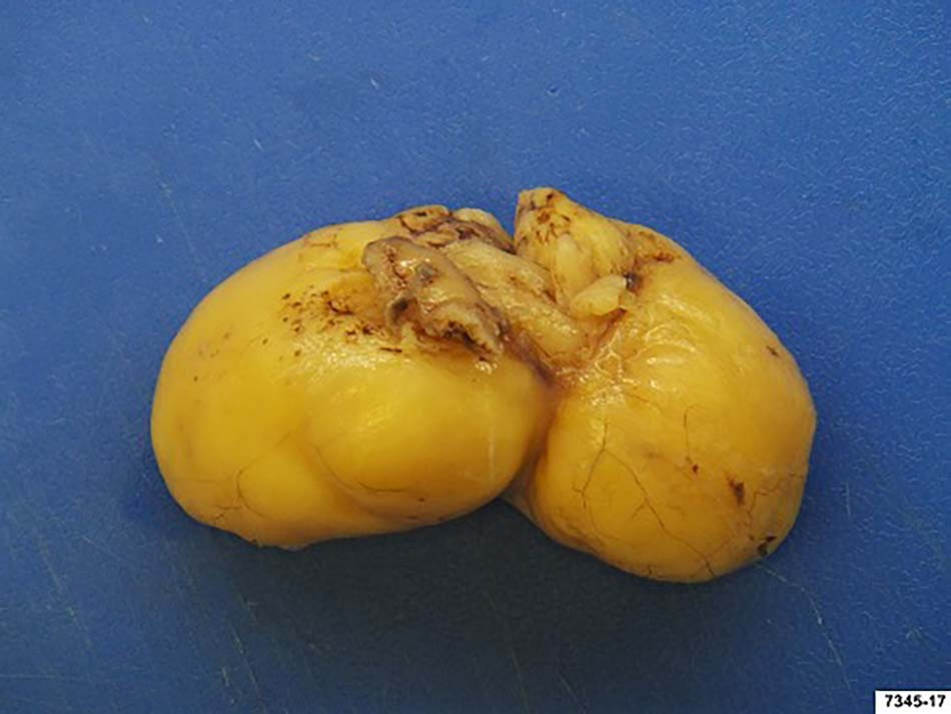
Surgical specimen: gastric lipoma with dimension of 80 × 35 × 35 mm.

We decided to perform a midline supraumbilical laparotomy. During the procedure, a large and mobile soft globular mass was palpated in the antrum (Fig. 3A), and anterior gastrotomy with tumour enucleation were performed (Fig. 3B, C and D). The postoperative recovery was uneventful, and the patient was discharged at postoperative day 6. Histopathological examination confirmed an 80 × 35 × 35 mm lipoma (Fig. 4). During the 18-month follow-up, the patient remained asymptomatic, disease-free and pleased with the outcome.

## DISCUSSION

Gastric lipomas are usually solitary, originating in the posterior wall of the antrum and are typically found in patients in their fifth and sixth decade of life [3, 10]. Malignant transformation is extremely rare, though simultaneous malignant lesions overlying the lipoma have been reported [8]. The differential diagnosis includes gastrointestinal stromal tumour, leiomyoma, leiomyosarcoma and adenocarcinoma [9].

An accurate preoperative diagnosis is based on endoscopic evaluation, EUS and CT examination [3, 10]. The endoscopy reveals smooth and yellowish submucosal mass, occasionally with areas of mucosal ulceration. The accurate identification of a gastric lipoma is aided by three classical endoscopic features that help to define gastric lipomas: (i) a “cushion sign”, an indentation of the mass when pressed with biopsy forceps; (ii) a “tenting sign”, the ability to separate the mucosa from underlying lipoma using biopsy forceps and (iii) a “naked fat sign”, when fat protrudes from the mass after multiple biopsies [9].

Abdominal CT is the imaging study of choice and highly specific. In the case of gastric lipoma, it strongly suggests the diagnosis by visualization of a well-circumscribed homogeneous mass with fat density ranging between −80 and −120 Hounsfield units. These values are considered pathognomonic and suggest a diagnosis of gastric lipoma, by falling in the expected value range for adipose tissue [4, 5]. EUS is another useful imaging study to confirm the hypothesis of gastric lipoma. By EUS, gastric lipomas appear as homogeneous and hyperechoic lesions from the third layer of the gastric wall [7, 10]. Our patient was diagnosed on endoscopy and confirmed with CT and EUS.

Because of pathognomonic appearance of gastric lipomas on endoscopy, EUS and CT scan, endoscopic biopsies is rarely needed. If a biopsy is needed, regular mucosa biopsies are usually inadequate to sampling because of the submucosal localization of the most lipomas, as it was the case on our patient. Alternatively, EUS guide-sampling can be used [3, 6, 10].

The treatment for gastric lipomas is not standardized and is mostly based in case reports.

Because of the benign nature and well-encapsulated feature of lipomas, gastric preserving can be a treatment option and related to tumour size: endoscopic resection when < 4 a 6 cm and surgery when > 6 cm, perhaps endoscopy has been used to submucosal resection of a giant lipoma with 9 cm [3, 7, 9, 10]. A laparoscopic approach can be considered taking into account its availability and patient characteristics [3, 9].

We believed that if an accurate preoperative diagnosis is established and malignancy is carefully ruled out, a gastric preserving surgery like enucleation should be the treatment of choice to avoid major morbidity associated to partial gastrectomy.

## CONFLICT OF INTEREST STATEMENT

None declared.
